# Endometrial Cancer Stem Cells: Role, Characterization and Therapeutic Implications

**DOI:** 10.3390/cancers11111820

**Published:** 2019-11-19

**Authors:** Gaia Giannone, Laura Attademo, Giulia Scotto, Sofia Genta, Eleonora Ghisoni, Valentina Tuninetti, Massimo Aglietta, Sandro Pignata, Giorgio Valabrega

**Affiliations:** 1 Department of Oncology, University of Torino, 10124 Torino, Italy; giulia.scotto@ircc.it (G.S.); sofia.genta@ircc.it (S.G.); eleonora.ghisoni@ircc.it (E.G.); valentina.tuninetti@ircc.it (V.T.); massimo.aglietta@ircc.it (M.A.); giorgio.valabrega@ircc.it (G.V.); 2 Candiolo Cancer Institute, FPO - IRCCS - Str. Prov.le 142, km. 3,95, 10060 Candiolo (TO), Italy; 3 Department of Urology and Gynecology, Istituto Nazionale Tumori IRCCS Fondazione G. Pascale Napoli, 80131 Napoli, Italy; laura.attademo@gmail.com (L.A.); s.pignata@istitutotumori.na.it (S.P.)

**Keywords:** cancer stem cell, endometrial cancer, target therapy

## Abstract

Endometrial cancer (EC) is the most frequent gynecological cancer. In patients with relapsed and advanced disease, prognosis is still dismal and development of resistance is common. In this context, endometrial Cancer Stem Cells (eCSC), stem-like cells capable to self-renewal and differentiation in mature cancer cells, represent a potential field of expansion for drug development. The aim of this review is to characterize the role of eCSC in EC, their features and how they could be targeted. CSC are involved in progression, invasiveness and metastasis (though epithelial to mesenchimal transition, EMT), as well as chemoresistance in EC. Nevertheless, isolation of eCSC is still controversial. Indeed, CD133, Aldheyde dehydrogenase (ALDH), CD117, CD55 and CD44 are enriched in CSCs but there is no universal marker nowadays. The most frequently activated pathways in eCSC are Wingless-INT (Wnt)/β-catenin, Notch1, and Hedghog, with a high expression of self-renewal transcription factors like Octamer binding transcription factor 4 (OCT), B Lymphoma Mo-MLV Insertion Region 1 Homolog (BMI1), North American Network Operations Group Homebox protein (NANOG), and SRY-Box 2 (SOX2). These pathways have been targeted with selective drugs alone or in combination with chemotherapy and immunotherapy. Unfortunately, although preclinical results are encouraging, few clinical data are available.

## 1. Introduction

Endometrial cancer (EC) is the most common gynecological tumor in developed countries. Rates for new diagnosis have been rising on average by 1% for each year over the last 10 years, with an incidence of 79 cases per 100,000 women in Europe and a median age at diagnosis of 62 years [1,2].

Known risk factors are early menarche, late menopause, polycystic ovary syndrome (PCOS), infertility, obesity and diabetes although genetic predisposition with presence of Lynch Syndrome is fundamental in patients with diagnosis before the age of 50 [3,4,5].

Historically, sporadic EC has been divided in two pathogenetic types, according to the so-called Bokhman’s dualistic model: estrogen-dependent endometrioid (Type 1) and estrogen-independent non-endometrioid carcinomas (Type 2) [6]. Almost 80% of EC type I patients had lower grade (G1 or G2) tumors, while 20% had high grade (G3) tumors [6]. They usually have low potential for lymphovascular invasion, high estrogen (ER) and progesterone receptors (PgR) expression, and a favorable prognosis [6]. Type II EC is characterized by prevalence of high grade (G3) (66% versus 34% low grade), high potential for lymphovascular invasion, low progesterone sensitivity, and a dismal prognosis [6].

Although Bokhman’s dualistic model is still relevant in clinical practice, more recently, the Cancer Genome Atlas (TCGA) has proposed a molecular classification defining four EC types with different clinical outcomes, on the basis of their overall mutational burden, specifically p53, polymerase episilone (POLE), and phosphatase and TENsin homolog (PTEN) mutations, microsatellite instability (MSI), and histology [7].

About one third of patients have localized cancer at diagnosis [1]. The prognosis for EC patients with early-stage or localized disease (stage I and II) is generally favorable. The majority of them can be successfully cured with surgery alone or followed by brachytherapy (BT) and/or external beam radiation therapy (EBRT) [8,9,10,11], with the addition of platinum-based chemotherapy in stage I high risk and in stage II patients [12]. The 5-year overall survival (OS) is 95% for stage I and 69% for stage II [1]. Nevertheless, about 13% of high-risk patients and 3% of low risk patients recur [13,14]. Prognosis for recurrent and advanced disease (stage III or IV) is poor, with 5-year OS in patients with metastatic disease rates ranging from 15 to 17% [1]. Standard first line treatments are a combination of carboplatin and paclitaxel and therapeutic options beyond first-line are limited, with low response rates to either single agent chemotherapy or endocrine therapy with medrossiprogesterone acetate (MPA) [12], due to the rapid onset of resistances. New drugs have been developed among which the checkpoint inhibitors showed interesting activity, leading to FDA approval of Pembrolizumab alone in MSI high tumors but also in combination with an angiogenesis inhibitor (Lenvatinib) in EC without MSI [15,16,17,18]. Nevertheless, new therapeutic approaches are still needed to significantly improve the prognosis of women with recurrent or advanced EC.

The basis of cancer stem cells (CSCs) theory is one of the most intriguing strategies to overcome drug resistance exploits stemness related pathways.

Historically, cancer research and therapies were based on the theory of clonal evolution, which stated that neoplasms derive from the sequential expansion of preexisting somatic mutations [19]. This theory leaves some open questions including the evidence of clonal heterogeneity of tumors [20], allowing the development of drug resistance [21]. More recently increasing evidence has led to the formulation of the so-called CSCs theory, which assumes that tumors, similarly to normal tissue, are composed by cells at different maturation stages including also CSCs. A CSC is a cell with stem-like properties, which has originally acquired the oncogenic mutation and gains the possibility to self-renew and to differentiate, being responsible for the generation of the entire neoplastic cell population and of their heterogeneity, drug resistance and invasiveness [22].

The first evidence supporting the CSC theory was derived from Virchow and Cohnheim studies [23]. These data were confirmed from a preclinical study published by Bonnet et al in 1997 demonstrating that cells capable to generate human AML (Acute Myeloid Leukemia) in xenograft models had proliferative capacities and the ability to differentiate and self-renew indicating that normal stem cells, rather than committed progenitor cells, were the target for the neoplastic transformation [24]. Since then, the presence of CSC has been reported in several different type of tumors including breast [25], colorectal [26,27,28], prostatic [29], central nervous system cancers [30], melanomas [31] and sarcomas [32].

Nevertheless, new data from high throughput sequencing, described new cancer models, among which the so-called big bang model. In this model, initiating cells grow producing several subclones with clonal alterations that are pervasive but not submitted to clonal selection while new subclonal mutations will be generated thanks to replication errors in smaller and smaller populations, conferring survival advantage and causing intratumor heterogeneity [33]. All these theories, far from being opposite, integrate and complete each other, describing and justifying presence of pervasive mutations but also tumor heterogeneity, drug resistance and above all plasticity. Indeed both CSC and differentiated cancer cells show plasticity, that is the capability of a phenotypic transition if stimulated [34]. Plasticity connects both CSCs and differentiated cells to microenvironment, with cells that move “up and down the hierarchy of differentiation” thanks to genomic instability and justifies once more heterogeneity [34].

During the last decades, several therapeutic strategies against CSCs have been attempted; some successful examples are represented by differentiation therapies for the treatment of acute leukemia [35,36,37], imatinib for gastrointestinal stroma tumors (GIST) [38] and sonidegib in basal cell carcinomas (BCC) [39]. Following these proofs of principle results, several inhibitors of signaling pathways [40,41], modulators of epigenetic mechanisms that regulate CSCs functions [42], or adoptive cell therapy targeting specific CSC antigens [43,44,45,46,47,48] are under clinical investigation.

The aim of this review is to describe the role of CSC in EC, their features, the most frequently involved pathways and how they could be targeted on the basis of preclinical evidence and clinical studies.

To do this, we performed a research on Pubmed and clinical trial.gov using the keywords “Cancer Stem Cells” and “Endometrial Cancer”.

## 2. CSCs in Endometrial Cancer: Role and Biomarkers

Hubbard et al. provided the first evidence of CSCs in ECs: small populations of cells obtained from EC samples generated tumors in immunocompromized mice. The morphology of daughter cells was similar to parental tumor, indeed they expressed cytokeratin, vimentin, estrogen receptor-α (ERα), and PgR [49] suggesting that a small population of cells is able to maintain features of parental tumor and to differentiate in vivo.

Self-renewal capacity, de-differentiation and ability to migrate from primary mass to blood vessels and other organs are the distinctive features of CSCs [50]. The self-renewal capacity is the property of CSCs to regenerate, leading to a subset of cells that aberrantly differentiates and survives despite the negative feedback signals of apoptosis [50]; this phenomenon seems to be at the basis of the tumor development and maintenance [51]. On the other hand ability to migrate but also to induce angiogenesis and to produce extracellular matrix plays a key role in EC metastasis [50]. Lastly CSCs are intrinsically chemoresistant, on the basis of their plasticity, playing a role in low response rate to chemotherapy in this disease [52].

To date, three theories have been postulated on the “origins” of endometrial CSCs.

The first hypothesis assumes that CSCs arise from normal/adult stem cell (SSC). The progressive acquisition of genetic mutations and epigenetic alterations may lead to CSCs transformation [53].

Current evidence suggests that CSCs could originate from differentiated tumor cells: the expression and release of “steamness” molecules seems to induce a de-differentiation program [54].

Finally, the somatic and differentiated cells of endometrium could represent the CSCs progenitor with acquisition of self-renewal and plasticity properties [52].

Regardless “origins”, an important issue is definition of biomarkers that can identify CSCs.

Different molecules were studied as markers of stemness in ECs [55]. To describe the main biomarkers, we performed research on Pubmed using the keywords “Cancer Stem Cells” and “Endometrial Cancer”, manually selecting papers on this topic. We excluded articles before 2010 and those not in English language.

CD117 (c-kit) is a cell-surface receptor tyrosine kinase type III that, if stimulated by Stem Cell Factor (SCF), induces cell replication, survival and differentiation and has been assumed to be a CSC marker for several tumors [56,57,58]. It has been shown that CD117^+^ EC cells showed a greater proliferative and colony forming capacity in a SCF-dependent manner. Indeed, using an anti-SCF antibody, the colony-forming activity decreased in vitro [59]. High CD117 expression was also recognized as an independent prognostic factor for OS [59]. CD44 and CD55 are others transmembrane proteins related to EC stemness. CD44 is an adhesion molecule implicated in process of invasion and metastasis and has been studies as marker of CSCs in ECs [60,61,62]. Indeed the spheres generated from EC cell lines showed CD44 expression [60] with evidence also of co-expression of CD44 and CD133 in endometrial CSCs [63].

Saygin et al. reported CD55, an intrinsic cell surface complement inhibitor, as highly expressed in endometrioid ovarian and endometrial CSCs and recognized that CD55^+^ cells were able to regulated self-renewal and cisplatin-resistance more than CD55^−^ ones [64].

All markers previously described are highly expressed in cells with in vitro stem cell properties while a “side-population” (SP) and CD133, listed below, are not only expressed in CSC in vitro, but also in cells transplanted in vivo.

Cluster of Differentiation (CD) 133, also known as prominin-1, is a member of pentaspan transmembrane glycoproteins, which specifically localize to cellular protrusions. Although the function of CD133 is not yet clear, a role in the organization of the cell membrane has been hypothesized [65]. Several authors have identified CD133 as a potential marker of CSCs in different solid tumors including brain [30], prostate [66], colon [27] and ovarian cancers [67,68]. Rutella et al. analyzed 113 tumor samples of EC and demonstrated that isolated CD133^+^ cells showed a more aggressive proliferation in vitro, a greater colony-forming ability and an higher resistance to cisplatin and paclitaxel compared with CD133^−^ cells [63]. Moreover, when serially transplanted into immunocompromized mice, CD133^+^ cells were capable of generating tumors characterized by a phenotype comparable with the one of the original tumor [63]. The role of CD133 as marker of tumorigenic potential in EC cells was confirmed by various works [61,62,69,70,71].

Several preclinical studies identified SP in cancer cell lines, defining a cellular phenotype capable of active draining fluorescent dye Hoechst 33342 outside cytoplasm [72]. SP cells are, indeed, characterized by a high expression of ABCG2 proteins, a super-family of ATP-binding cassette (ABC) transporters. These proteins facilitate efflux of proteins, lipids, ions, and above all, an anti-cancer drug, inducing chemoresistance [73,74].

Kato et al. [75] proved that a SP subpopulation is represented in human EC cells and rat endometrial cells with activated KRAS gene. These cells showed proliferative and self-renewal capacity, enhancement of migration, tumor formation and chemoresistance [75,76,77,78]. Nakamura et al. identified an SP fraction from EC cell lines with CSC features that co-expresses CD133. SP cells phenotype and CD133 expression seem to be independent markers of stemness and their co-expression is correlated to higher proliferation rate and tumor formation [77].

When transplanted in nude mice, SP cells generate large and invasive tumors composed by both tumor cells and stromal-like cells producing extracellular matrix. SP derived tumors express higher levels of vimentin, α-smooth muscle actin, collagen III and fibronectin than non-SP derived tumors, all markers of mesenchymal differentiation [75,76].

Indeed, Epithelial-to-mesenchymal transition (EMT) is an essential process during early embryonic development as well as a mechanism involved in cancer progression [79]. The EMT pathway allows malignant cells to metastasize from a primary tumor by losing epithelial features and obtaining mesenchymal phenotype [79]. These evidences suggest that endometrial cancer SP cells are also capable of undergoing EMT.

Lastly Aldehyde dehydrogenase 1 (ALDH1) is one of the 19 different enzymes involved in oxidation of Aldeide [80]. It shows high activity in early stages of stem cell differentiation [81,82]. EC cells that express high ALDH1 are more tumorigenic, invasive and resistant to cisplatin than low expressing ALDH1 cells. High expression of ALDH1 correlates with a worse prognosis in EC patients (*p* = 0.01 for Overall Survival (OS)) [83]. Kitson et al. compared CD133^+^ cells to high ALDH1 cells, showing that the latter had greater CSCs activity and higher expression of genes involved in EMT than CD133^+^ cells [84].

A recent work conducted by Tabuchi and colleagues demonstrated heterogenic CSCs populations in EC [85]. They isolated two distinct types of clones from one patient tumor sample, according to their growth pattern: the sphere clones (S clones), that grow forming spheres, resistant to chemotherapeutic agents but less tumorigenic and leukemia-like clones (LL clones), that grow separately, like leukemia cells, sensitive to chemotherapy but highly tumorigenic [85]. These results reveal that in the same sample there are different CSCs, suggesting that probably there is not a unique biomarker that can identify CSCs in EC and that different biomarkers could be expressed by different colonies in the same tumor.

This leads also to absence of consensus on the optimal technique to separate CSC from differentiated cells. Indeed, different approaches have been developed, among which fluorescence activated cell sorting, individuation of SP, with and without the use of Hoechst 33342, and use of a serum free suspension cultivation with the addition of growth factors. Specifically, this medium drives to formation of microsphere, proliferation of CSCs and elimination of differentiated cells [86].

## 3. Activated Pathways in Endometrial CSCs

Stemness in EC is regulated by different pathways among which Notch, Wnt, and Hedgehog pathways seem to have the most relevant roles.

Notch signaling pathway regulates cell development and differentiation. It is highly conserved in most mammals and implicated in many mechanisms that control multiple cell differentiation processes during embryonic and adult life [87].

Notch signaling cascade keeps the undifferentiated state of cells but can also induce cell differentiation under appropriate stimulation. Several evidences showed that dysregulated Notch signaling is involved in tumorigenesis and cancer development being overexpressed in breast cancer, gastric cancer, colon cancer and pancreatic cancer [88].

Musashi-1 is an RNA binding protein involved in Notch-1 signaling pathway: Götte et al. showed an increased expression of Musashi-1 protein in endometriosis and endometrial carcinoma and expression of Small interfering-RNA (siRNA), able to inhibit Musashi- 1, in EC cells, reduces cell proliferation inducing also apoptosis [89].

Nanog is a key multidomain homeobox transcription factor, placed on the chromosome 12, which contributes to the maintenance of the undifferentiated state of pluripotent stem cells. Nanog downregulation induces cell differentiation. Several studies link Nanog overexpression to EC cells [49,90]. Transcription factors octamer-binding transcription factor 4 (Oct4), SRY-Box 2 (SOX2), Transcription Factor 3 (Tcf3) and Forkhead Box D3 (FoxD3) contribute to the regulation of Nanog expression. OCT-4 and SOX-2 were found in endometrial CSCs and linked to the potential of self-renewal capacity [49,90].

The Wnt pathway (or Wnt/β-catenin pathway), known as the canonical pathway, is involved in several processes that are essential for embryonic development and normal adult homeostasis [91].

The canonical pathway requires Wnt ligand binding to Frizzled receptors to initiate the intracellular signaling via β-catenin nuclear translocation. The phosphorylation of a negative regulator of the destruction complex, Dishevelled (Dvl), causes the recruitment of Axin, inhibiting its interaction with other components of the destruction complex. Thus, ß-catenin accumulates in the cytoplasm and translocates to the nucleus, where it activates the transcription of Wnt related genes as cyclin-D1 and MYC (MYC Proto-Oncogene, Basic Helix Loop Helix (BHLH)Transcription Factor) [91].

Alterations in Wnt signaling have been related to the development of many different malignancies, including acute myeloid leukemia (AML), breast and gastrointestinal cancers [92]. Kusanoki and collegues have shown that in endometrial cancer downregulation of Wnt signals leads to inhibition of proliferation and migration of endometrial CSC [76].

As described above, EMT is a process related to stemness in ECs. In the work of Yusuf et al. Secreted Protein Acidic and Rich in Cysteine (SPARC) a protein involved in extracellular matrix synthesis is expressed in poorly differentiated endometrioid adenocarcinoma with enhanced fibronectin expression and promotes migration and formation of tumor stroma [78].

In a recent work by Lu and colleagues the expression of SPARC-related modular calcium-binding 2 (SMOC–2), a member of SPARC family, was found to be higher in endometrial CSCs [93]. High Levels of SMOC-2 positively correlate with the expression of CD44 and CD133. Moreover, they demonstrated that SMOC-2 is involved in activation of Wnt/ β-catenin pathway and is related to cisplatin and paclitaxel resistance [93].

Also, (PTEN-Phosphoinositide 3 kinases (PI3K)-Protein kinase B (AKT)-Mammalian target of rapamycin (mTOR) pathway plays a role in maintaining stemness through the upregulation of EMT inducers such as B Lymphoma Mo-MLV Insertion Region 1 Homolog (BMI-1) , that downregulates PTEN expression, Enhancer of zeste homolog 2 (EZH2) that participate to histone methylation, Slug and Snail, that downregulate E-caderin expression [94].

Hedgehog signaling pathway acts on embryonic cells growth and differentiation. Its downstream effector is a transmembrane protein, Smoothened (SMO) that activates Glioma (Gli) transcriptional factor 1 and expression of target genes at the end. Their activity is regulated by the membrane receptor patched (Ptch1) [95]. Feng and colleagues showed that expression of Sonic Hedgehog and SMO is significantly higher in ECs if compared with normal Endometrial epithelium and endometrial hyperplasia [96].

Lastly, not only genetic pathways but also epigenetic modulators and metabolic preferential pathways are important in maintaining cancer stemness. For example the expression of high levels of micro-RNA 21 in ECs have been shown to downregulate PTEN expression, leading to EC cells proliferation [97]. A preferential oxidative pathway with a higher glucose uptake and a lower lactate production could differentiate CSC from differentiated tumor cells [98].

In conclusion, several pathways are involved in EMT and self-renewal properties of endometrial CSCs: these pathways are strictly interconnected and some of them are under investigation as possible targets of therapy.

**Figure 1:**
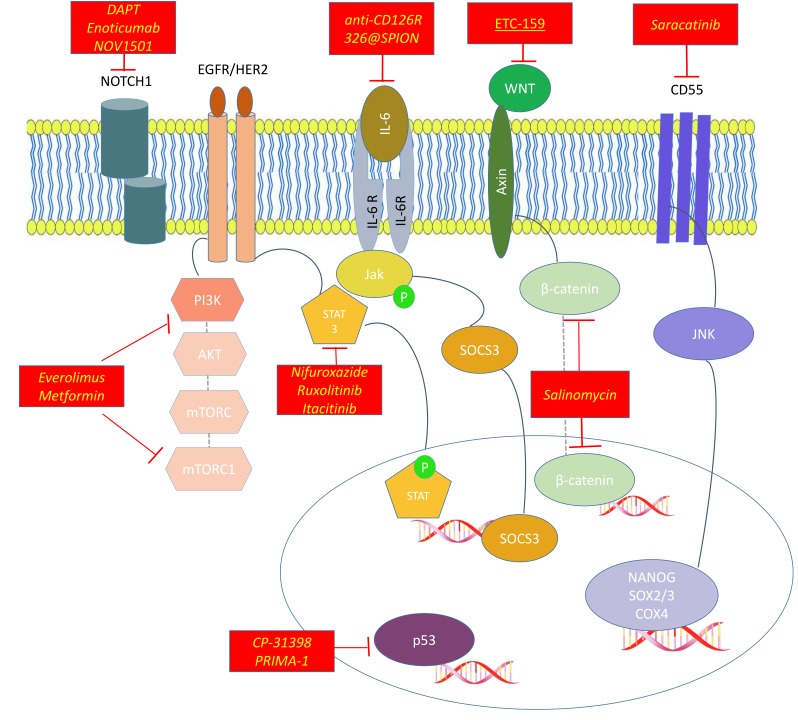
Activated pathways in Endometrial Cancer Stem Cells (CSCs) and their inhibitors.

For the most important CSC pathways and how we could target them, see Figure 1.

The most frequently activated pathway in CSCs are:

Hedgehog signaling pathway, with its downstream effectors SMO and Gli1; its inhibitors are sonidegib and cyclopamine.
PI3K/AKT/mTOR Complex (mTORC)pathway, inhibited by everolimus and metformin.
Wnt/β-catenin pathway, inhibited by salinomycin and ETC-159.
NOTCH pathway whose inhibitors are DAPT ((N-[N-(3,5-difluorophenacetyl)-L-alanyl]-S-phenylglycine t-butyl ester))), Enoticumab and NOV1501 (also ALB001).

Other agents target transmembrane proteins like CD55 (saracatinib) or transcription factors involved in maintening stemness like upstream and downsteam inhibitors of Signal transducer and activator of transcription 3 (STAT3)(ruxolitinib, nifuraxozide and itacitinib), inhibitors of B Lymphoma Mo-MLV Insertion Region 1 Homolog BMI-1 (like PTC-028) or modulators of p53 (like CP-31398 and PRIMA-1).

## 4. Agents Targeting CSCs in EC: Preclinical Development

As described before, CSCs play a main role in tumor progression and chemoresistance. Following those evidences many new molecules have been developed, in order to target distinctive pathways of stemness.

Interesting targets are membrane proteins, for example CD55 or CD117, that also regulate SOX2, NANOG and OCT4 expression, maintaining self-renewal and induce drug resistance. Saygin and colleagues demonstrated that inhibition of CD55 with saracatinib produces cisplatin resensitization of both ovarian and endometrial endometrioid cell lines while Zhang and colleague demonstrated that cisplatin resistant CSCs are sensitive to imatinib [59,64]. Another target evaluated in preclinical setting was ALDH1. Inhibition of ALDH activity with disulfiram and N, N Diethylaminobenzaldehyde (DEAB) reduces proliferation of spheroids composed by CSCs [99]. On the other hand, an antibody directed against Epithelial membrane protein-2 (EMP2) (a protein involved in tumor migration and neoangiogenesis) reduces tumor formation and growth in ALDH1^+^ cells and indirectly downregulates ALDH1 expression although the precise mechanism of action is not clear [100]. Another mechanism that induces chemoresistance and CSC survival is autophagy which is probably related to ALDH expression. Xiaomin Ran and colleagues demonstrated that with the addition of an autophagy inhibitor [3-methyladenine (3-MA) or Cloroquine (CQ)] to EC cultured spheroids, there is a reduction in cell growth and self-renewal capability [101].

As described above NOTCH signaling is responsible of CSC viability and proliferation. Preclinical works demonstrated that selected miRNAs inhibit this pathway. For example miRNA134 inhibits proliferation in vitro and in vivo of CD44^+^/CD133*^+^* subpopulation in type II EC, suppressing Protein O-glucosyltransferase 1 (POGLUT1) expression, that is a regulator of NOTCH signaling [102]. MiRNA-34a acts directly against NOTCH1 reducing tumor formation in Patients derived Xenografts (PDXs). It also reduces expression of vimentin, EMT and invasiveness of HEC-1-B cells [103]. This paved the way for development of NOTCH inhibitors, among which DAPT (N-[N-(3,5-difluorophenacetyl)-L-alanyl]-S-phenylglycine t-butyl ester)) showed to synergize with an Epithelial growth factor receptor (EGFR) inhibitor (AG1478) in HEC-1A and IK cell lines reducing cell viability [104].

Most preclinical studies focused on Wnt/β-catenin pathway inhibition. Liu and colleagues demonstrated that Membrane Associated Ring-CH-Type Finger 7 (MARCH7) is a key protein in EMT and cancer stemness. It indeed increases Vimentin and Snail levels in EC cells. Its inhibitor miR-27b-3p reduces the migration and invasion of Ishikawa cells and downregulates EMT related proteins [105].

A molecule that targets this pathway is Salinomycin, a monocarboxylic polyether antibiotic. It inhibits proliferation of eCSC from human and rat lines and reduces levels of fibronectin, BCL2 and cyclin-D that are downstream Wnt pathway [76]. Salynomicin acts also inhibiting ABC proteins, overcoming thus drug resistance mediated by these efflux pumps in CSCs [106]. These pumps have been exploited also by Yaguchi and colleagues. They indeed demonstrated that ABC proteins are induced by estrogens and controlled via Sirtuin 1/cAMP response element binding protein (SIRT1/CREB) signaling pathway. Its inhibitor reserpine reduced E2-induced cell proliferation and viability [107].

Also, inhibition of Hedghehog pathway could be an interesting strategy. Targeting Hedgehog with cyclopamine reduces EC cells viability between 56% and 67% in two different ER^+^ cell lines at 72 h, with a downregulation of cyclin D1 and N-myc in HHUA cells [96].

Among transcription factor, BMI-1 play a key role in EMT and PI3K/AKT but also in Notch and Hedgehog downstream pathways. Indeed inhibition of BMI-1 with a si-RNA, induces a reduction of number of CSCs (with a lower expression of SOX2 and Oct4) and of cancer invasiveness [108]. PTC-028, a second-generation inhibitor of BMI-1, decreases invasion of ECs and activates caspase-dependent apoptosis [109].

Targeting Signal transducer and activator of transcription 3 (STAT3) demonstrated to be a resourceful tool. Both inhibiting upstream pathways like Interleukin 6 (IL-6) pathway with an antibody directed against the CD126 receptor or with a miRNA (mir326 bound to a nanoparticle 326@SPION) against G Protein-Coupled Receptor 91 (GPR91) pathway both using small molecule directed against STAT3 (nifuroxazide) and against Janus kinase 1 (JAK1- ruxolitinib) reduces in vitro and in vivo ALDH^+^ HEC1A cells proliferation and sensitize CSCs to cisplatin [110,111].

Inhibition of other transcription factors acting on embryonic development was studied in preclinical setting, among these miRNA194 inhibits SOX3 expression, a high-mobility group box protein that is involved in EMT and stemness of EC. It directly binds 3-untranslated region (UTR) of SOX3, reducing growth and metastasis of EC tumorspheres [112].

Other drugs inhibit CSCs growth, although their activity involves also non-CSC specific pathways, such as drug metabolism, epigenetic or hormone dependency.

Being known that estrogens stimulate growth of ER^+^ CSC spheroids with upregulation of IL6, IL1B an IL18 [99], Guy and colleagues showed that MPA decrease CD133^+^ populations both in ER^+^/PgR^+^ but also in ER^−^/PgR^−^ cell lines. Moreover there was a reduction in CD133^+^ cells in samples of patients who responded to MPA treatment although number of samples was small (only 4 patients) and they all showed ER positivity [113]. 

Histone deacetylase inhibitors (like Sodium Butyrate) reduce viability of EC cell lines with an enhanced activity on CSCs through DNA damage and overproduction of Reactive oxygen species (ROS) [114].

Inhibition of PI3K pathway both with a si-RNA acting against BMI-1 as described above [108] or with Metformin reduces CSCs activity. Metformin is active on CSCs at a high dose (0.5–1 mM) both reducing cell proliferation and CSCs related genes expression but it doesn’t act on self-renewal capacity [84]. Moreover patients’ derived adipocyte media reduces cell response to metformin suggesting that microenvironment can influence responsiveness of CSCs to this drug [84].

Modulators of p53, like CP-31398 and PRIMA-1, reduce viability and growth of ALDH-expressing cells, suggesting an activity of these compounds on stemness related pathways [99].

For the most important drugs acting on CSCs see Figure 1.

## 5. Agents Targeting CSCs in EC: Clinical Trials and New Perspectives

As for clinical activity, several early phase studies are evaluating drugs targeting CSCs pathways in EC patients.

Regarding agents directed against PI3K pathway their possible role in the treatment of EC patients has been widely investigated in clinical studies. They have showed interesting activity in pretreated ECs patients. Phase II trials evaluated mTOR inhibitors alone and registered a 4% of Response rate (RR) with temsirolimus ev [115], up to 5% with everolimus [116] and between 8.8 and 11% of RR with ridaforolimus [117,118]. Most common Adverse events (AEs) included anaemia, mucositis, fatigue, hypercholesterolemia and hyperglycemia. On the basis of crosstalk with ER pathway, combinations were studied with the aim of improving activity of these compounds. The first attempt with a combination of temsirolimus , megaestrol acetate and tamoxifen was unsuccessful with no increase in RR compared to temsirolimus alone but a higher percentage of venous thromboembolisms [119].

On the other hand, in a phase II trials, patients receiving a combination of letrozole and everolimus achieved a RR of 32% with a manageable toxicity profile leading to its inclusion as a therapeutic option for advanced EC in National Comprehensive Cancer Network (NCCN) guidelines [120]. Activity of metformin in EC was also evaluated in clinical trials, retrospective series and a systematic meta-analysis suggesting that patients using metformin had a higher OS compared to controls (Hazard Ratio (HR) = 0.82; Confidence Interval (CI): 0.70–0.95; *p* = 0.09) [121]. On the basis of these promising results, Soliman et al evaluated a combination of everolimus, letrozole and metformin achieving a 29% of Partial Response (PR) (14 over 48 patients evaluable) with a safe profile; diarrhea, anemia, fatigue, nausea, mucositis, hypertriglyceridemia, and high transaminase were the most common adverse events [122]. Other ongoing studies are evaluating even more powerful combinations that target not only the PI3K/AKT pathway and Endocrine Receptors but also cell cycle [123] (NCT01797523, NCT03008408).

Among strategies that directly target CSCs pathway, agents acting on Notch have been studied. Delta like 4(DDL4) is a Notch ligand specifically expressed in endothelial cells. In a phase I trial Enoticumab, a DLL4 inhibitor, was evaluated among 54 patients with advanced solid tumors showing a safe profile with G3 nausea and vomiting as dose limiting toxicities (DLTs) and initial data of activity with a Response Rate RR of 5% and a stable disease in 16% of patients [124]. Another DLL4 inhibitor (NOV1501, also ABL001) is under investigation also in EC (NCT03292783).

Signature program is an ongoing platform of basket trials among which solid tumors with alteration of Hedgehog pathway could receive Sonidegib, an inhibitor of SMO (NCT02465060) [125]. Indeed Sonidegib showed a safe profile in a phase I trial with increased serum creatinine as the most frequent G3/G4 Adverse events (AE) (17% of patients) [39] and is approved for the treatment of adult patients with locally advanced BCC [126].

Among strategies developed to target Wnt pathway, inhibitors of Porcupine, an acetyltransferase involved in secretion of Wnt ligands, have been studied in early phase clinical trials [127]. ETC-159 is a Porcupine inhibitor. It shows manageable side effects in interim analysis of a phase I trial in which this agent was evaluated in combination with denosumab in 5 patients. Dysgeusia (62%), fatigue (37%), weight loss (37%), back pain (37%) and headache (37%) were the most frequent AEs [128]. An ongoing trial is evaluating ETC-159 in endometrial cancer alone or in combination with pembrolizumab (NCT02521844). This trial is one of several attempts to exploit immune microenvironment to target also CSCs on the basis of immune suppression induced by stem related pathways such as β-catenin in the Wnt signaling [129].

The few studies available suggest a safe profile of these drugs however data on activity are still awaited.

For a list of main ongoing trial targeting CSCs in ECs see Table 1.

## 6. Discussion

CSCs play a main role in cancer progression and drug resistance. Their expression in EC correlates with tumor aggressiveness and chemoresistance. Different biomarkers have been developed to detect and isolate CSC, both transmembrane proteins and dynamic tests or enzymes like ALDH1. Among these, EMT plays a role both as a marker of stemness and an activated pathway. Other pathways involved in cancer stemness are Hedgehog signaling pathway, PI3K/AKT/mTOR Complex (mTORC) pathway and NOTCH pathway. These pathways have been exploited therapeutically and early phase trials suggest that both NOTCH inhibition with Enoticumab and Hedgehog inhibition with Sonidegib could be active against endometrial CSC. Nevertheless, combination of drugs that exploit PI3K related pathways seems to be the most promising strategy. 

Several questions are still open:

- Which is the best biomarker to detect CSCs?
- Is there a dependency on a specific pathway?
- Which is the most promising strategy to target CSCs?
- Probably CSCs heterogeneity justifies the presence of multiple biomarkers [85]. Indeed, CD133 and ALDH1 are the most studied and their expression correlates with prognosis and aggressiveness [63,83]. On the other hand markers of EMT [60] and expression of efflux pumps of ABCs family are certain markers of CSCs suggesting that the definition of a cluster of biomarkers could be the best strategy to detect CSCs in EC.
- CSCs related pathways are multiple and crosstalking, suggesting that there is no strict dependency on a specific pathway. Wnt-βcatenin [76], Notch [89] and Hedgehog [90] are the most important but also other signaling cascades implied in multiple biological mechanism like ER [113] and PI3K/AKT pathways [94] are involved in maintaining endometrial CSCs with a main role played also by epigenetic modulators.
- Nowadays we have few clinical data on drugs targeting CSCs. Certainly, most early phase studies showed that these drugs are safe but data on activity are lacking. Crosstalk and redundant pathways maintaining stemness are probably intrinsic mechanisms of resistance in several cancers and next therapeutic strategies could consider combination of drugs. The addition of drugs against CSC pathways to chemotherapy could be a tool to overcome resistance in ECs. An even more promising combination is the one of checkpoint inhibitors with drugs targeting CSCs above all in inflamed tumors among which MSI-high EC is one of the most interesting example [15,130]. Another powerful strategy could be treatment with chimeric antigen receptor-modified T (CART) cells, that recognize specific antigens, inducing T cell activation and survival, thus resulting in antitumor activity [44]. Preclinical works suggest that CSC-targeted CART cells against CD133 are effective in peritoneal carcinomatosis from gynecological cancers PDXs [131] but there is no ongoing study with CART133 including patients with ECs.

Evaluating toxicities is one of the main challenges for the future, remembering that CSCs could be targeted also in an indirect way. Among indirect strategies PI3K/AKT inhibitors and hormonal agents showed interesting activity in EC [120].

## 7. Conclusions

In conclusion CSCs are crucial in cancer initiation, metastasis and above all, drug resistance but appear also to be druggable. Nevertheless, diagnostic biomarker validation is essential for the development of successful, biology oriented therapeutic strategies. Ongoing early phase trials are evaluating safety of these approaches paving the way for development of a new and fascinating precision medicine in a setting of poor valuable therapeutic options.

## Figures and Tables

**Table 1:** Ongoing clinical trials targeting Cancer Stem Cells (CSCs) in Endometrial Cancer.

Description	Condition	Line of Therapy	Primary Endpoint	Phase	Status	Trial Identifier
Targeted Therapy Directed by Genetic Testing (Pi3k inhibitors, AKT inhibitors, Hedgehog Antagonist)	Advanced solid tumors	≥2 line	ORR	II	Recruiting	NCT02465060
DKN-01 +/− Paclitaxel	EC, OC or Carcinosarcoma	≥2 line	ORR, toxicity, pharmacokinetic	II	Recruiting	NCT03395080
ETC-159 +/− Pembrolizumab	EC, OC or colorectal cancer	≥2 line	% AE	I	Active not recruiting	NCT02521844
COTI-2 (Against p53) +/− cisplatin	EC or Other solid tumors	na	DLT, MTD, RP2D	I	Recruiting	NCT02433626
Everolimus, Letrozole, and Metformin	EC	≤3 line	CBR	II	Active not recruiting	NCT01797523
Ribociclib+ Everolimus + Letrozole	EC	≤ 3 line	DLT/CBR	I/II	Recruiting	NCT03008408
Gedatolisib + Palbociclib	Solid tumors	NA	MTD, RP2D, %AE	I	Recruiting	NCT03065062
Cyclophosphamide + Metformin + Olaparib	EC	≥2 line	RP2D	I/II	Notyetrecruiting	NCT02755844
AZD2014(MTOR inhibitor) + anastrozole	ER^+^ EC	≥2 line	% AE, 8weeks PFS	I/II	Recruiting	NCT02730923
NOV1501 (anti DLL-4)	Solid tumors	NA	DLT	I	Recruiting	NCT03292783
Itacitinib (anti Jak 3) (INCB039110) and/or Pembrolizumab	Solid tumors	NA	Toxicity	I	Active not recruiting	NCT02646748

Legend: AEs: Adverse Events, CBR: Clinical Benefit Rate; DLTs: Dose Limiting Toxicities; EC: Endometrial Cancer; ER^+^: Estrogen Receptor positive; MTD: Maximum Tolerated Dose; NA: not available; OC: Ovarian Cancer; ORR: Overall Response Rate; PFS: Progression Free Survival; RP2D: Recommended Phase II Dose.
